# Evaluation of Record Keeping in Exodontia Department of Punjab Dental Hospital, Lahore: A Clinical Audit

**DOI:** 10.7759/cureus.33645

**Published:** 2023-01-11

**Authors:** Omer Bin Zahid, Ajwa Rehman, Muhammad Shoaib, Mehak Bilal, Asmi Shaheen, Muhammad Hassan Jamil, Shamza Siddique

**Affiliations:** 1 Dentistry, de'Montmorency College of Dentistry, Lahore, PAK; 2 Orthodontics, de'Montmorency College of Dentistry, Lahore, PAK

**Keywords:** oral surgery records, dentistry, record keeping in dentistry, exodontia, record keeping

## Abstract

Introduction: An audit was conducted in the exodontia department of Punjab Dental Hospital, Lahore, to assess the quality of records being kept by the undergraduate students in their third and final year, who form a major chunk of the workforce in the hospital, working in the mentioned department. The main objective behind this exercise was to improve the standards of record keeping and bring them in line with the standards practiced around the world, ultimately resulting in better patient care.

Methodology: This audit was undertaken while keeping in view all the necessary steps of a successful clinical audit. Initially, 150 records were randomly obtained from undergraduates of both third and fourth years and evaluated against a modified CRABEL score, which grades the records on a scale of 100. The results of this part of the audit were shared with the batches that were doing their clinical rotation in exodontia at the time of this audit, and a teaching session was conducted on better record-keeping standards. Following this, a repetition of the previous audit was undertaken to complete the audit cycle.

Results: The most commonly omitted component in the records in the initial audit was the patient complaint closely, followed by proper medical history and supervisor signatures. In the following, ‘reaudit’ compliance was seen to be improved, and all the components of record-keeping less commonly being omitted except medical history and date.

Conclusion: A more comprehensive patient record keeping is possible with proper intervention and inculcation of record-keeping awareness in the undergraduate course, especially in the clinical years.

## Introduction

Audits and quality improvement projects are paramount to practicing quality medicine and improving the standards of care in medicine. It is especially relevant in dentistry at a public sector hospital where a large volume of patients is treated daily. A proper record of a dental procedure performed is of the utmost importance in the clinical setting and for the welfare of both the patient and the dentist. It ensures the streamlining of the treatment plan too [1].

“Care record standards exist to improve the safety and quality of health and social care, in particular, to ensure that the right information is recorded correctly, in the right place, and can be accessed easily by any authorized person who needs it, wherever they are” [2]. 

Clinical practice during the years spent at a medical or dental school is a crucial practice for the development of a habit of proper record keeping. A proper record helps reevaluate the treatment plan for a patient, makes the treatment easier in the subsequent visits, aids in medicolegal situations, and provides valuable insight into the patient's illness which may also serve as an object in the teaching of the students. The ability of a dental practitioner to maintain a proper dental record is, at the very least, an ethical obligation, if not a legal one. Dental records are an essential component serving as an information source for dentists and patients in medicolegal, administrative, and financial functions within the general practice for quality assurance and audit [3].

According to the guidelines provided by the NHS in the United Kingdom, the record form of a new patient must include his/her name, date of birth, phone no., address, payment receipt, medical history, the reason for attendance, and the dentist's findings on examination and radiographic evaluation [4]. In a previous audit conducted at a multispecialty hospital in Karachi, the standard of record-keeping was found to be lacking [5]. This is concerning on many levels since it not only indicates the lack of awareness about the importance of proper record keeping but also indicates a declining level of patient care. Punjab Dental Hospital, an allied hospital of de’Montmorency College of Dentistry, is the largest tertiary care hospital in the most populous metropolis of Punjab, the most densely populated province of Pakistan. Conducting this exercise at the undergraduate level is aimed to determine whether an emphasis on the importance of record keeping, structured as a part of undergraduate clinical training, will make a difference in the quality of records maintained by these students when they become clinicians practicing independently since the quality of records maintained by dentists has previously been found to be of insufficient quality [6,7]. This problem needs to be addressed from the very start. However, certain gaps in the curriculum and training structure during dental education leave much to be desired [8]. With this audit, the authors aim to assess the quality of the records kept by the undergraduate students of a tertiary care dental hospital in Pakistan and to improve the quality of patient care in the hospital.

## Materials and methods

Certain guidelines have been set by the Royal College of Surgeons of England for maintaining medical records [9]. Centered around these guidelines, a scoring system has been developed by Crawford, Beresford, and Laferty and named CRABEL, an amalgamation of their names [10]. Student records were randomly selected from the logbooks of students in the third and final year who had rotated in Surgery Unit I of Punjab Dental Hospital from April 2022 to September 2022 since there was no adequate record of undergraduate work kept by the hospital administration. A total of 150 records were pulled for examination, and seven records were deemed unsuitable. One hundred forty-three records were ultimately used for this audit. Most of these records were written on the backs of the hospital payment slip instead of a proper record-keeping form. These records were investigated and scored against the modified CRABEL scoring system used previously for the evaluation of dental records [11]. A maximum score of 100 can be attained by a record. Each of the components was given a score of 10, which was deducted from the total of 100 if that component was missing. Five points were deducted where the component was present, but some part of it was illegible due to any reason. 

After this initial scoring, a presentation was delivered by the lead author and the supervisor to a group of around 25 students of the third and final year who were working in the Surgical Unit I at the time. The findings of the initial audit were shared, and special emphasis was placed on the upkeep of international standards of record-keeping and their importance in the dental practice and the use of a proper record-keeping form instead of the back of the payment slip. After this, the records kept by students for the next four weeks were examined and scored against the same CRABEL scoring system explained in Table 1. 

**Table 1:** CRABEL scoring system used in this audit.

Component	Score
Patient Name	10
Hospital Case Note Number	10
Date	10
Department	10
Accurate Medical History	10
Patient Complaint	10
Procedure Performed	10
Treatment Plan	10
Student’s Signature	10
Supervisor’s Signature	10

## Results

**Figure 1:**
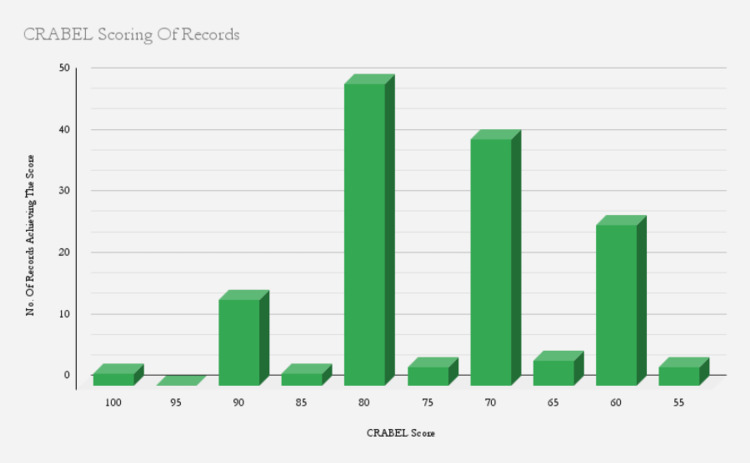
Record scores in the initial audit.

Results of the initial audit are shown in Figure 1, where the scores of the 143 records examined are represented. 

The lowest score achieved was 55, and the highest was a perfect score of 100. However, most of the records were found to fluctuate between the range of 60 and 80, with 80 being the most commonly obtained score.

**Figure 2:**
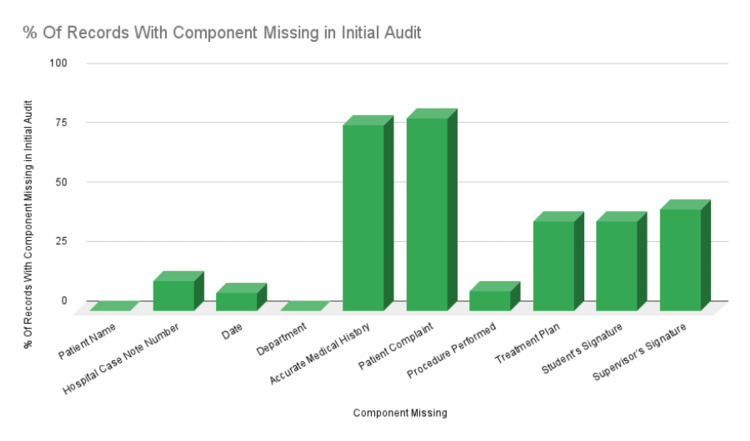
Percentage of records with components missing in the initial audit

Also, the percentage of omission of individual components is represented in Figure 2.

**Figure 3:**
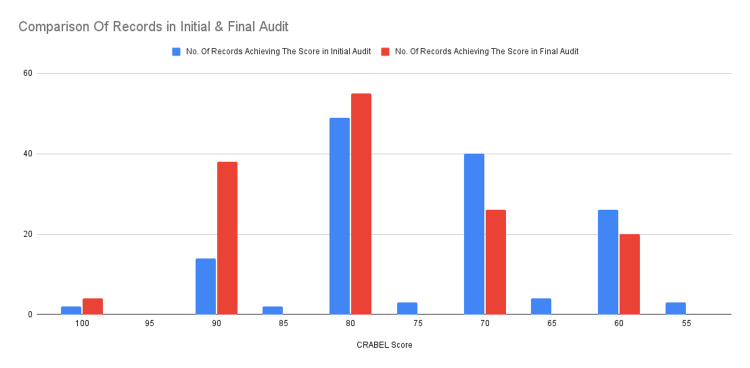
Comparison of record scores in the initial and final audit

From the above figures, it can be determined that the most commonly omitted component in the initial dental records examined was the patient complaint that was missing in 116 records, closely followed by an accurate medical history that was missing in 112 records. The supervisor’s signature was also found missing in a significant number of records (61). The results of the second audit showed improved record-keeping standards and the use of the proper record-keeping form that was present in the logbooks of the students. The highest score achieved was 100, and the lowest score was 60. The overall CRABEL score improved, as shown in Figure 3, most likely due to the students' knowledge of the increased scrutiny of records and their evaluation against a standard.

**Figure 4:**
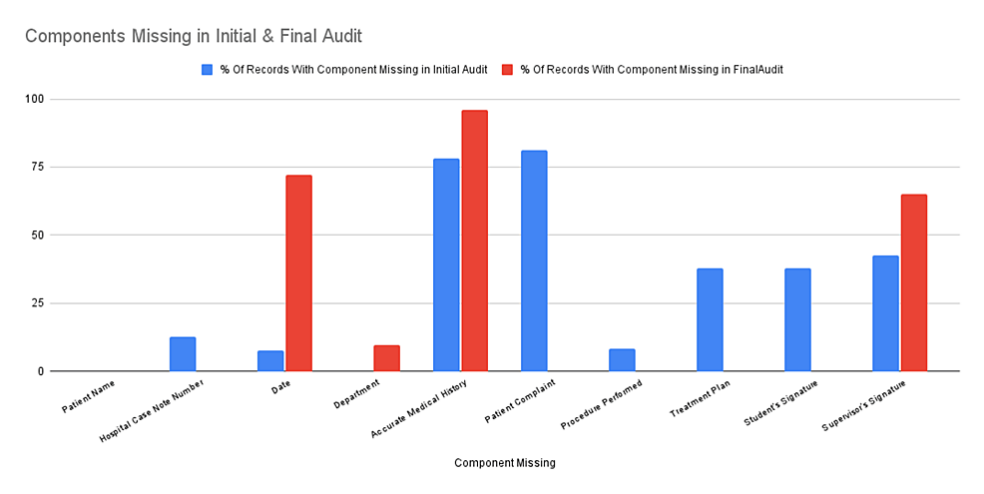
Comparison of the percentage of components missing in the initial and final audits

The most commonly omitted component of record-keeping in the final audit was the accurate medical history, which had changed from the initial audit, followed by the date and signature of the supervisor, shown in Figure 4.

## Discussion

Dentistry is a unique subject due to a large amount of hands-on training and direct interactions of the undergraduate students with the patients, and since these students will be expected to practice independently after a few years and previous studies by McGleenon E., Morison S have found that supervisors expressed dissatisfaction in the treatment planning done by students [12]. This highlights the importance of maintaining standardized records for undergraduate students since it will have a direct effect on their lives as a clinician, especially when they are practicing independently, as proper maintenance of patient records will ensure the truth of the doctor and proves that the treatment was carried out properly [13].

**Figure 5:**
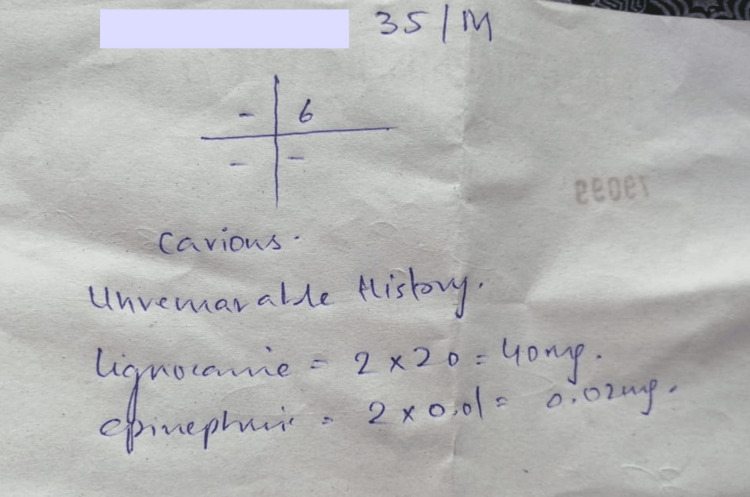
An example of a record kept on the back of a payment slip

Although the score of dental records of students at de'Montmorency College Of Dentistry was found to be higher than reported by some other dental colleges (taken from a similar audit done by Basit A, Shah SM, Jameel RA, et al. at Hamdard University Dental Hospital), it was still found to be subpar in the initial audit, especially the use of a blank piece of paper to keep records (an example of that ‘payment slip record’ is shown in Figure 5) instead of the proper form available in their log books. This is concerning, especially for the students in their final year, because it is generally accepted that they have, by now, a working knowledge of the basics of dentistry [14]. They are soon to graduate and work independently as dentists without supervision, so proper record-keeping is a highly essential skill that needs to be emphasized at this stage. However, after highlighting the shortcomings and the lecture on the importance of record-keeping, scoring improved significantly, and the problem of using a blank paper for record-keeping was also rectified when the students were aware of the increased scrutiny. 

This change, while a good omen, does not negate the fact that scores of medical history, date, and supervisor signature had worsened in the final audit. Most of the medical history written by the students was either incomplete or was found insufficient to perform dental surgery even after the intervention. A proper lecture on medical history taking and noting it down should be conducted at regular intervals to plug this glaring hole in the standard of record keeping by the students. 

This re-audit was conducted soon after the intervention, which may be the reason why the average CRABEL score of the records improved while the lecture was still not forgotten by the students. These lectures and evaluation of records by the teachers should be made a regular practice to ensure the upkeep of the standards since patient records are the permanent account of a patient and assures that the patient's needs were met [15].

Since Punjab Dental Hospital is a teaching institute affiliated with de’Montmorency College of Dentistry, such change can be affected quite easily.

Further, we acknowledge that the students were aware that there was increased scrutiny of the records, which may have contributed to better scores; however, this is not deemed a bad thing since the ultimate purpose of this audit was to improve patient care and to improve the standards of record keeping.

Limitations of this audit are that factors affecting the documentation ability of the doctors were not examined. Furthermore, the quality of record-keeping can also be influenced by the type of record-keeping form provided in each hospital or private setup. However, adherence to the standards of record-keeping is the pathway to ethical conduct in dentistry and medicine as a whole. 

## Conclusions

The results of this audit are to be shared with the relevant department so they can understand the shortcomings of the students and how to rectify them. Consideration should be given to incorporating the lectures on record-keeping as a part of every clinical rotation and to educate the students on the use of a proper record-keeping form instead of writing on the back of a payment slip. The supervisors should scrutinize every record before signing it to address the problems and the falling standards of record keeping. This audit may be repeated when these changes have been implemented.
